# Genome-Wide Identification and Characterization of the mTERF Gene Family in Spinach and the Role of *SomTERF5* in Response to Heat Stress

**DOI:** 10.3390/plants14111570

**Published:** 2025-05-22

**Authors:** Ziyue Sun, Li Li, Yaqi Liu, Yanshuang Liu, Gaojian Li, Yueyue Li, Qingbo Yu, Meihong Sun, Xiaofeng Xu

**Affiliations:** 1 Shanghai Engineering Research Center of Plant Germplasm Resources, Shanghai Collaborative Innovation Center of Plant Germplasm Resources Development, College of Life Sciences, Shanghai Normal University, Shanghai 200234, China; sun_ziy@163.com (Z.S.); 17701799317@163.com (L.L.); liuyaqi001208@163.com (Y.L.); yanshuangliu@yeah.net (Y.L.); ligj1126@163.com (G.L.); 2021031283@nefu.edu.cn (Y.L.); yuqing9860@shnu.edu.cn (Q.Y.); 2 State Key Laboratory of Tree Genetics and Breeding, School of Forestry, Northeast Forestry University, Harbin 150040, China

**Keywords:** Spinach, SomTERF family, *SomTERF5*, heat stress

## Abstract

Spinach (*Spinacia oleracea* L.), a globally consumed, nutrient-dense vegetable, contains diverse vitamins and minerals. However, elevated temperatures can constrain yield by interrupting leaf development and photosynthetic efficiency. The mitochondrial transcription termination factor (mTERF) family, which regulates organellar gene expression, plays crucial roles in plant growth and photosynthetic regulation. Thus, characterization of the spinach mTERF (SomTERF) family is critical for elucidating thermotolerance mechanisms in this crop. In this study, we systematically identified 31 *SomTERF* genes from the spinach genome, which are distributed across five chromosomes and nine unassembled genomic scaffolds. Subcellular localization predictions indicated that these proteins predominantly target chloroplasts and mitochondria. Conserved domain analyses confirmed that all SomTERF proteins possess canonical mTERF domains and ten conserved motifs. Phylogenetic clustering segregated these proteins into nine distinct subgroups (I–IX), with significant divergence observed in gene copy numbers among subgroups. Cis-element screening identified an abundance of heat-, cold-, and hormone-responsive motifs within *SomTERF* promoter regions. Notably, seven members (including *SomTERF5*) exhibited pronounced enrichment of heat shock elements (HSEs). Organ-specific expression profiling revealed preferential leaf expression of these seven genes. Comparative RT-qPCR in heat-sensitive (Sp73) and heat-tolerant (Sp75) cultivars under thermal stress demonstrated genotype-dependent expression dynamics. Functional validation of *SomTERF5* was achieved through cloning, and transgenic *Arabidopsis* overexpressing *SomTERF5* showed significantly enhanced thermotolerance, as evidenced by improved survival rates following heat treatment. Yeast two-hybrid (Y2H) assays further revealed physical interaction between SomTERF5 and SopTAC2. This study provides a comprehensive foundation for understanding mTERF-mediated developmental regulation and advanced molecular breeding strategies for developing heat-resilient spinach varieties.

## 1. Introduction

Spinach (*Spinacia oleracea* L.), a nutritionally rich green vegetable, is distinguished by its cold tolerance but marked susceptibility to heat stress, which substantially constrains its cultivation and productivity [1]. Consequently, the breeding of heat-resistant spinach varieties holds significant economic and practical value. While both the heat-sensitive spinach variety (Sp73) and the heat-tolerant variety (Sp75), along with Sp75’s high-quality chromosome-scale reference genome, have been documented [2,3], the molecular mechanisms underlying their differential responses to heat stress remain largely elusive.

Photosynthesis is highly sensitive to temperature fluctuations [4]. Precise transcriptional regulation coordinates the spatial and temporal expression of photosynthesis-related genes by integrating light signaling, environmental stress, and metabolic feedback, ensuring that plants efficiently capture light energy, fix CO_2_, and adapt to environmental changes under different conditions [5,6]. Transcriptomic analysis of spinach leaves has identified 896 unique genes involved in the heat stress response, which are critical for signal transduction, reactive oxygen species (ROS) homeostasis, transcriptional regulation, and protein stability under heat stress [2]. However, the functional roles of these heat stress-responsive genes in spinach remain largely uncharacterized. Comparative analysis of thermos-responsive proteins between Sp73 and Sp75 revealed significant differences in photosynthetic inhibition, ROS scavenging capacity, chlorophyll and carotenoid biosynthesis, and soluble sugar content [7]. Under heat stress, Sp73 exhibited reduced photosynthetic acclimatization and osmotic homeostasis capacities, as well as altered activities of superoxide dismutase (SOD) and other antioxidant enzymes compared to Sp75. These findings provide valuable insights into the mechanisms underlying heat tolerance in spinach.

Chloroplasts are not only the sites of photosynthesis but also serve as hubs for numerous fundamental intermediary metabolic reactions in higher plants. As semi-autonomous organelles, chloroplasts evolved from cyanobacterial ancestors [8] and possess their own genome. The accurate expression of these genetic materials is crucial for plant growth and development. Chloroplast gene expression is a highly complex process that encompasses transcription, post-transcriptional processing, and translation [9,10,11]. Given the limited coding capacity of chloroplasts, these processes rely on a multitude of regulatory factors localized to the chloroplast but encoded by the nuclear genome. Thus, coordinating the expression between the chloroplast and nuclear genomes is essential for proper plant function. The mitochondrial transcription termination factor (mTERF) family was first discovered in metazoans [12]. These mTERF proteins contain approximately 30 amino acids in tandem with mTERF domains, and they can be divided into four categories [13,14]. In vertebrates, the mTERF family consists of only four members, and six mTERF members named MOC1-MOC6 [15,16] have been identified in *Chlamydomonas reinhardtii*. However, *Arabidopsis* initiative genome analysis revealed 35 *mTERF* genes in the genome [17], suggesting that the *mTERF* gene family has expanded significantly. Comprehensive investigations of other plant genomes have revealed 31 mTERF members in the maize genome [18], 17 members in the peanut genome [19], 48 members in the rice genome [20], and 28 members in the tomato genome [21]. These analyses further support the view that the *mTERF* gene family has significantly experienced expansion in higher plants, and they also suggest that the family has important roles in plant growth and development.

In vertebrates, members of the mTERF family are localized in the mitochondria [12,22,23]. The pioneer work on the functional investigation of this family was human mTERF1 [22]. This mTERF1 protein was initially thought to promote the transcription termination of heavy strands (HSs) in human mitochondrial genes. Available data show that the functions of the mTERFs family act as positive and negative regulators of mitochondrial transcription in invertebrates and mammals and regulate the translation of mitochondrial genes [22]. In contrast, members of this family are predominantly localized to chloroplasts and/or mitochondria in higher plants [24,25]. Their functions are associated with both mitochondrial and chloroplast gene expression, including transcription/post-transcriptional regulation [26,27], transcription termination [28,29], and translation [27,30]. In *Arabidopsis*, the mutation of *mTERF4* (*RUG2*) induces thermos-sensitivity, with mutant plants maintaining normal development at 16 °C but showing complete growth cessation under 26 °C conditions [31]. We previously found that mTERF8 regulates transcription termination of the chloroplast gene *psbJ*, maintaining photosystem II efficiency [29]. To date, systematic genome-wide identification and characterization of the members of the spinach mTERF gene family have yet to be reported.

In this study, we identified 31 SomTERF members within the spinach genome, which are distributed across five chromosomes. Molecular evolutionary analysis classified these members into nine subgroups (I–IX), with significant variations in subgroup size. Analysis of cis-acting elements revealed that seven members possess heat stress and hormone response elements in their promoter regions. RT-qPCR experiments demonstrated significant differences in the expression patterns of these members under heat stress in two spinach varieties, Sp73 and Sp75. Notably, *SomTERF5* was cloned and overexpressed, resulting in enhanced heat tolerance in transgenic lines. Yeast two-hybrid assays confirmed the interaction between SomTERF5 and SopTAC2. These findings provide a valuable foundation for the development of heat-tolerant spinach varieties.

## 2. Results

### 2.1. Phylogenetic Relationships Between Members of the Spinach mTERF Family and Other Species

To determine the SomTERF family of spinach, we downloaded the genome from the genomic data of SpinachBase (http://www.spinachbase.org/; accessed on 5 June 2023) [3]. We used the 35-member protein sequences from *Arabidopsis* as queries to search the spinach genome database using BLASTp, setting a cutoff value of 1E^−100^ for the expected value (e-value). To confirm the conserved domain, we submitted the candidate SomTERFs to the NCBI web CD-search tool (https://www.ncbi.nlm.nih.gov/Structure/bwrpsb/bwrpsb.cgi; accessed on 12 June 2023). A total of 31 SomTERF members were identified from the spinach genome through a hidden Markov model (HMM) [32] and structural domain analyses (Table S1). Based on protein homology with *Arabidopsis* mTERF members [2], these 31 proteins were designated as SomTERF1 to SomTERF31. The analysis revealed that the coding regions of these *SomTERF* genes ranged from 639 to 2244 base pairs, with amino acid sequence lengths varying from 212 (SomTERF12) to 747 amino acids (SomTERF2). Predictions of subcellular localization using Predotar [33] and WolFPSORT (https://wolfpsort.hgc.jp; accessed on 15 June 2023) indicated that 13 SomTERF proteins were localized in mitochondria (42%), 15 were localized in chloroplasts (48%), and the remaining 3 were localized in other cellular locations (10%).

**Figure 1:**
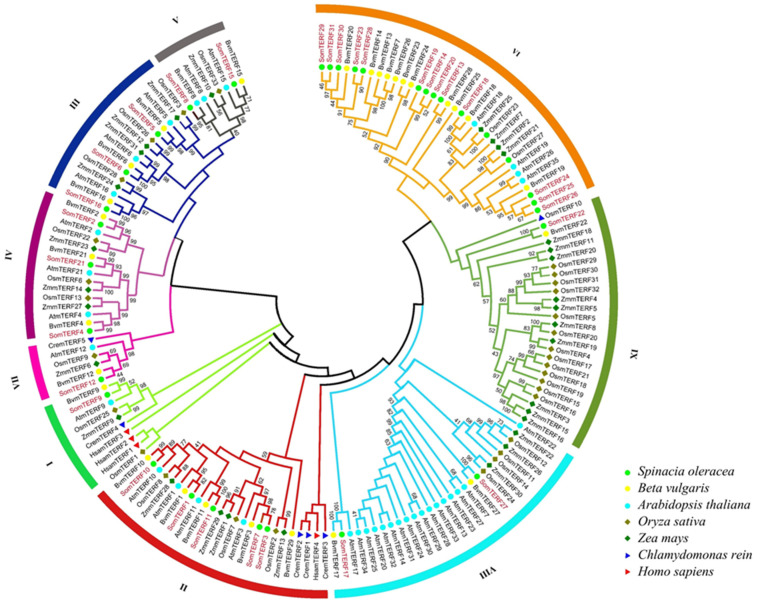
Phylogenetic analysis of plant mitochondrial transcription termination factor families (mTERFs) from spinach and six other representative plants. A total of 168 mTERFs were analyzed based on complete amino acid sequences from spinach (*Spinacia oleracea*), sugar beet (*Beta vulgaris*), *Arabidopsis thaliana*, rice (*Oryza sativa*), maize (*Zea mays*), *Chlamydomonas reinhardtii*, and humans (*Homo sapiens*). Each group is highlighted in a different color.

To investigate the phylogenetic relationships of the spinach *mTERF* gene family with those of other species, a phylogenetic tree was constructed (Figure 1). This tree comprised 168 amino acid sequences of mTERF proteins, including the 31 identified members in spinach. Within the spinach species, 31 members were identified, while the sugar beet (*Beta vulgaris*) contained 29 members, *Arabidopsis* contained 35 members, rice contained 33 members, maize contained 31 members, humans contained 4 members, and *Chlamydomonas reinhardtii* contained 5 members. Phylogenetic analysis demonstrated that all mTERFs could be classified into nine distinct groups (Groups I–IX), with variations in the number of SomTERFs in each group. Group VI contained 13 SomTERF members, while Groups III, VII, and VIII each contained only 1 SomTERF member. Notably, SomTERF2 and SomTERF21 in Group I clustered distinctly with their sugar beet homologues, BvmTERF2 and BvmTERF21, respectively. Additionally, homologous mTERFs from monocotyledonous rice and maize were grouped into another cluster, suggesting a close association between the distribution of the spinach SomTERF family and species affinities. Phylogenetic analyses indicate that these SomTERFs exhibit a high degree of conservation across both monocots and dicots, consistent with comparative genomic analyses and their evolutionary relationships.

### 2.2. Analysis of Gene Structure, Functional Structural Domains, and Conserved Motifs of SomTERF Family Genes

**Figure 2:**
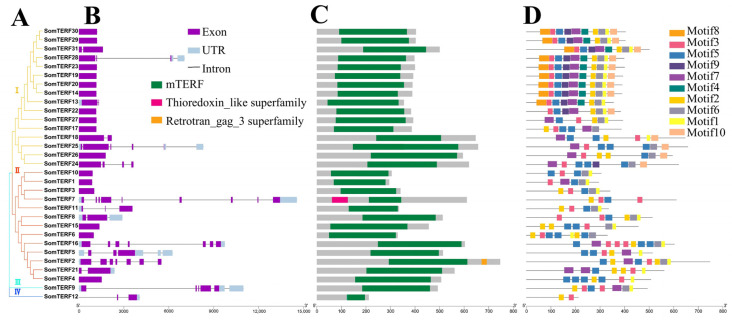
SomTERFs’ phylogenetic tree, gene structure, conserved motifs, functional domains, and modification sites. (**A**) The neighbor-joining method was used for the phylogenetic tree. (**B**) Exons, introns, and UTR structures in SomTERFs are illustrated. The gene structure includes exons, introns, and untranslated regions (UTRs). The gray box indicates UTR, the purple box indicates exons, and lines indicate introns. (**C**) Functional domains are presented. (**D**) Conserved motifs of SomTERFs are shown with distinct colored patterns.

Phylogenetic analysis indicated that spinach SomTERF proteins could be categorised into four groups: Group I included 16 SomTERFs, Group II included 13 SomTERFs, while Groups III and IV contained only one SomTERF each, specifically *SomTERF9* and *SomTERF12*, respectively (Figure 2A). To further explore the structural diversity of SomTERFs, exon–intron structures were analyzed. The results revealed that 16 members had no introns, four members contained one intron (*SomTERF31*, *SomTERF13*, *SomTERF18*, and *SomTERF21*), while the remaining members contained two or more introns, with *SomTERF7* exhibiting up to eight introns. Furthermore, members of Group I displayed similar structures and were predominantly devoid of introns, whereas the majority of Group II members contained introns (Figure 2B).

Protein structural domain analysis confirmed that all 31 members contained the mTERF structural domain. Additionally, SomTERF2 contained the Retrotran_gag_3 superfamily structural domain, and SomTERF7 contained the Thioredoxin_like superfamily structural domain (Figure 2C). To investigate the conserved motifs of SomTERFs, the MEME website [34] (accessed on 13 July 2023) was employed for analysis. A total of ten motifs were identified, with SomTERF12 exhibiting the fewest motifs, containing only two (motif2 and motif3) (Figure 2D). Most members of Group I contained all ten motifs, while the majority of Group II members possessed seven motifs. The arrangement of motifs among SomTERFs in each group exhibited a high degree of similarity.

### 2.3. Analysis of Cis-Acting Elements of the Spinach mTERF Family

**Figure 3:**
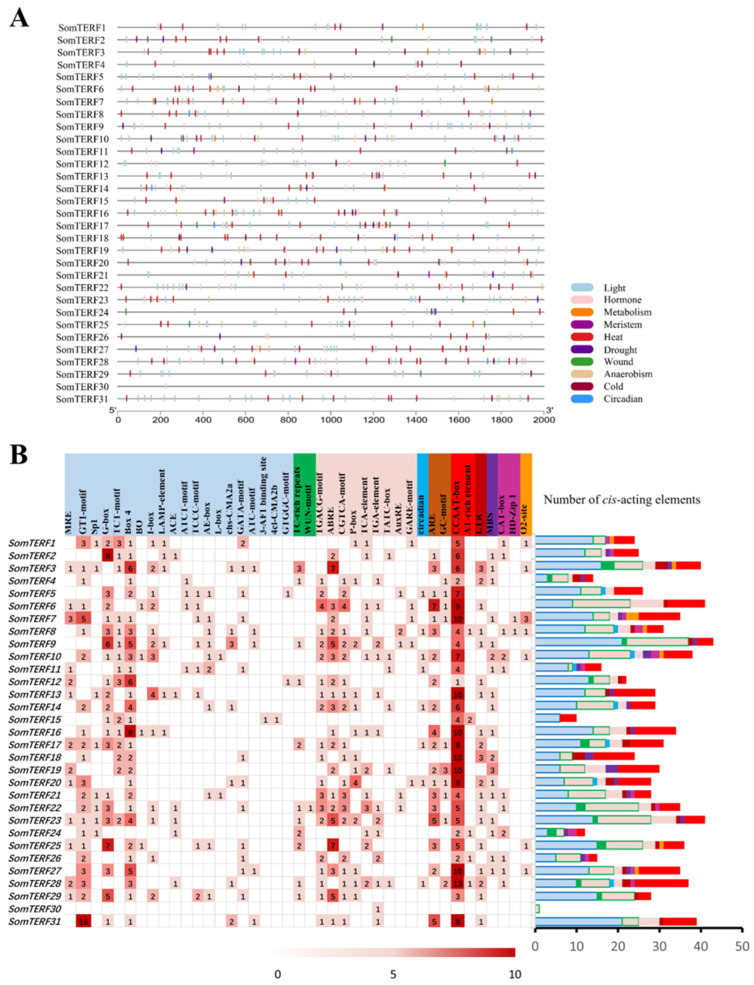
Analysis of the *cis*-acting elements in the promoter regions of *SomTERF* genes. (**A**) The *cis*-acting elements’ distribution in *SomTERF* promoters. (**B**) The names and numbers of *cis*-acting elements in *SomTERF* promoters. The heatmap and color columns in the grid indicate the number of cis-acting elements. Abbreviations: ABRE: ABA-responsive element; ARE: anaerobic-responsive element; LTR: low-temperature-responsive element; MBS: MYB-binding site; MRE: Myb recognition element; O_2_-site: regulation of zeinolysin metabolism; CAT-box: meristematic tissue expression; HD-Zip 1: fenestrated chloroplasts.

To explore the potential functions of *SomTERFs*, cis-acting elements in the promoter regions of seven *SomTERF* genes were predicted, and the distribution of these elements related to stress responses is schematically depicted herein (Figure 3). These 31 identified *SomTERF* promoter sequences (2000 bp upstream of the start codon) were submitted to the PlantCARE website [35] (accessed on 16 July 2023) for prediction, revealing the presence of *SomTERF* promoters in 10 categories of stress response elements (Figure 3). Among them, *SomTERF* members responsive to heat (CCAAT-box and AT-rich), light (GTI-motif, G-box, Box4, and MRE), phytohormones (ABRE, TGACG-motif, and CGTCA-motif), drought (MBS), and cold (LTR) were predominant. Notably, heat- and light-responsive elements were identified, with 194 heat-responsive elements and 27 cold-responsive elements present among the 31 *SomTERFs*. All members, except *SomTERF30*, contained multiple heat-responsive elements, with members such as *SomTERF3* (6), *SomTERF5* (7), and *SomTERF17* (8) exhibiting a significant number of heat-responsive elements. These findings suggest that *SomTERFs* play an important role in responding to abiotic stresses.

### 2.4. Expression Analysis of the SomTERF Family in Different Spinach Organs

**Figure 4:**
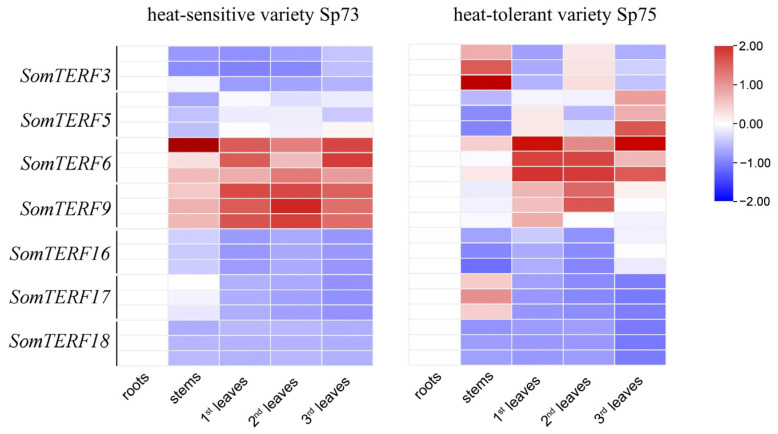
*SomTERFs* expression in 2 spinach varieties. Analyzed using RT-qPCR in heat-sensitive Sp73 and heat-tolerant Sp75. Results shown based on the 2^−ΔΔCt^ method, and relative gene expression levels were normalized using the expression level of *SoARF* [36]. Heatmap from log_2_ transformed data, with red/blue shown for up/down-regulated genes.

To understand the organ-specific expression of the seven *SomTERFs* identified as potential regulators of heat tolerance in spinach, organ-specific expression analysis was conducted. Total RNA was extracted from roots, stems, and three pairs of true leaves of the heat-sensitive variety Sp73 and the heat-tolerant variety Sp75 at the three-leaf stage. RT-qPCR of the seven *SomTERF* genes potentially regulating heat tolerance in spinach was performed. As shown (Figure 4), in the heat-sensitive variety Sp73, *SomTERF6* was highly expressed in stems and the third pair of true leaves, while *SomTERF9* exhibited the highest expression in the first and second pairs of true leaves. In the heat-tolerant variety Sp75, *SomTERF3* had the highest expression in stems, whereas *SomTERF6* was most highly expressed in the three pairs of leaves. Notably, the expression levels of all seven *SomTERF* genes were comparatively higher in the true leaves of both spinach varieties than in other plant parts, suggesting that these *SomTERF* genes are predominantly expressed in the leaves of spinach.

### 2.5. Expression Analysis of SomTERF Family Genes Under Heat Stress

**Figure 5:**
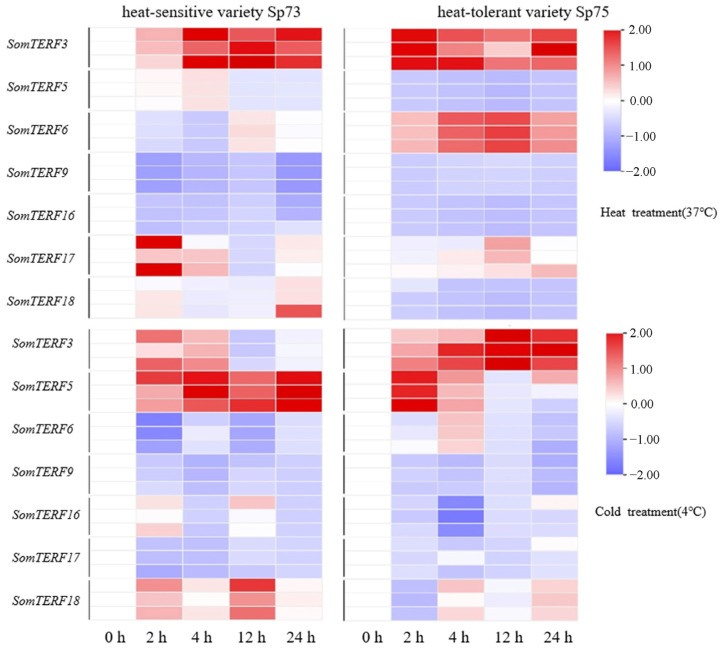
Expression profiles of *SomTERFs* in Sp73 and Sp75 leaves under temperature stress. Expression levels after heat (37 °C) or cold (4 °C) treatments for 0, 2, 4, 12, and 24 h. Calculated using the 2^−ΔΔCt^ method; relative gene expression levels were normalized using the expression level of *SoARF*. Heatmap from log_2_ transformed RT-qPCR data, with red/blue shown for up/down-regulated genes.

Extreme temperatures are known to inhibit seed germination and reduce plant growth and reproduction. In this study, 194 heat-responsive cis-acting elements were predicted in the promoters of *SomTERFs* (Figure 3). To investigate the role of *SomTERFs* in spinach heat tolerance, total RNA was extracted from spinach leaves subjected to different heat-tolerant materials, temperatures, and treatment durations, followed by RT-qPCR analysis. The results indicated that the expression of all seven *SomTERF* members was significantly altered (Figure 5). In the heat-sensitive variety Sp73, *SomTERF3* and *SomTERF6* exhibited up-regulated expression with increasing heat treatment (37 °C) duration compared to the normal condition (0 h). *SomTERF3* displayed the most pronounced up-regulation, while *SomTERF5* and *SomTERF9* showed a downward trend. In the heat-tolerant variety Sp75, *SomTERF3* and *SomTERF6* also demonstrated increased expression with prolonged heat treatment, whereas *SomTERF5* experienced a more substantial decrease. Under cold stress (4 °C), *SomTERF5* expression was significantly up-regulated in Sp73, while the expression levels of *SomTERF3*, *SomTERF16*, and *SomTERF18* initially increased before decreasing. In Sp75, *SomTERF3* expression was significantly up-regulated, while *SomTERF5* and *SomTERF6* exhibited a trend of increasing followed by decreasing expression, and *SomTERF9* showed a significant decrease in both varieties. The contrasting expression patterns of *SomTERF5* and *SomTERF17* in the two spinach varieties suggest that they may play important roles in the heat resistance process.

### 2.6. Subcellular Localization of SomTERF5 and SomTERF17

**Figure 6:**
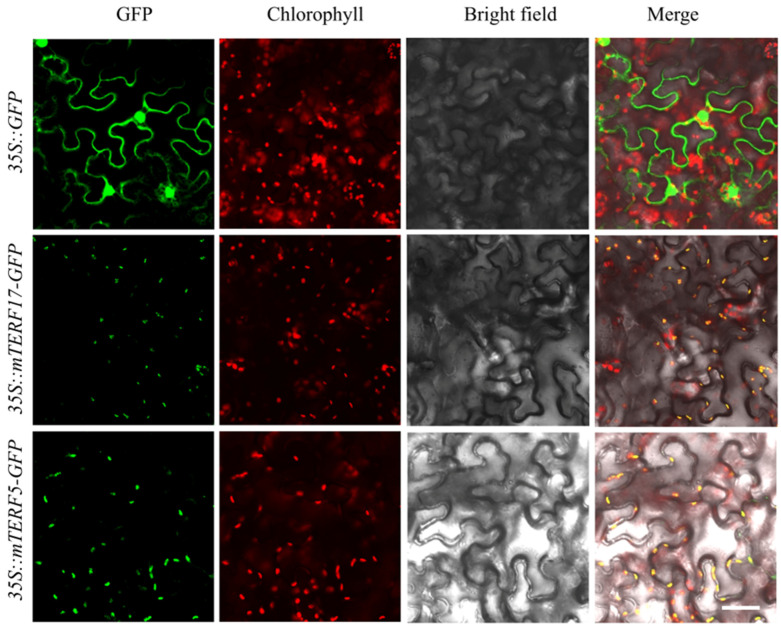
Subcellular localization of SomTERF5-GFP and SomTERF17-GFP in tobacco (*Nicotiana benthamiana*) leaves. 35S::GFP as the control. Proteins expressed by *Agrobacterium* in tobacco cells. GFP signals observed 48 h post-infection. Scale bar = 100 μm.

The subcellular localization of SomTERF was assessed using tobacco leaves. *SomTERF5* and *SomTERF17* are each fused to the 3′ end of the *GFP* reporter gene in a readable frame, regulated by the *CaMV 35S* promoter. The recombinant proteins SomTERF5-GFP, SomTERF17-GFP, and GFP alone were transiently expressed in tobacco leaf epidermal cells. Observation through confocal microscopy revealed that the green fluorescent protein (GFP) fluorescence of SomTERF5 and SomTERF17 was distributed in the chloroplasts of tobacco cells, overlapping with the autofluorescence of chlorophyll (Figure 6). The results indicated that both SomTERF5 and SomTERF17 proteins were localized in chloroplasts.

### 2.7. Thermotolerance Analysis of SomTERF5 in Arabidopsis

To study the function of *SomTERF5* in heat tolerance, we first obtained the *Arabidopsis mTERF5* (homologue of *SomTERF5*) mutant for co-treatment. The T-DNA insertion mutant of *mTERF5* (*mterf5*; Figure S1) was obtained from the AreShare mutant library (https://www.arashare.cn/index; accessed on 5 August 2023). To analyze the T-DNA insertion site of this mutant, PCR amplification and sequencing were performed. The results confirmed that the T-DNA was inserted into the exon of the *mTERF5* gene (Figure S1). Furthermore, RT-qPCR showed that *mTERF5* gene expression was significantly down-regulated in the *mterf5* mutant compared to the WT (Figure S1), confirming that *mterf5* is an *mTERF5* knockout mutant.

**Figure 7:**
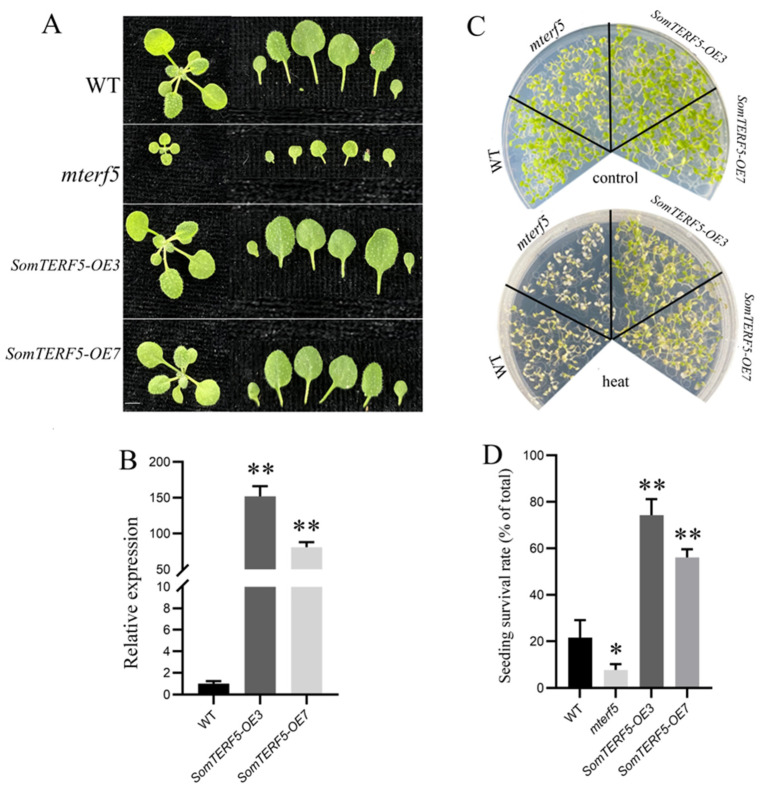
Thermotolerance analysis of the wild-type (WT), *mterf5* mutant, and *SomTERF5*-overexpressing *Arabidopsis* seedlings. (**A**) Leaf area comparison among the WT, *atmterf5*, and overexpressing lines. (**B**) RT-qPCR for *SomTERF5* expression in T2 to validate overexpression. (**C**) Phenotypes on 1/2 MS at 22 °C or after 8-day heat. (**D**) Survival rates after heat. Bars: mean ± SD. Data are shown as the means ± SD; *n* = 3; * *p* < 0.05 and ** *p* < 0.01 based on Student’s *t*-test.

The *mterf5* mutant exhibited a leaf-dwarfing phenotype (Figure 7A). To investigate the effect of *SomTERF5* on growth and development during the seedling stage, comparisons were made between the *mterf5* mutant, overexpression of *SomTERF5* (*SomTERF5-OE3* and *SomTERF5-OE7*), and WT plants. It has been shown that 4-week-old lines with overexpression of *SomTERF5* exhibit larger leaves and an early shoot phenotype compared to the WT under 20 °C conditions (Figure 7A). RT-qPCR further confirmed that the expression levels of *SomTERF5* in the *SomTERF5-OE3* and *SomTERF5-OE7* lines were 152-fold and 81-fold higher than those in the WT (Figure 7B). To assess the impact of *SomTERF5* overexpression on heat tolerance, seedlings of WT, *mterf5* mutant, and *SomTERF5* overexpressing lines were subjected to 44 °C for 2.5 h at 5 days of age, followed by survival rate assessment. Under normal conditions (22 °C, 16 h light/20 °C, 8 h dark), the survival rates of the WT, *mterf5* mutant, and these two *SomTERF5* overexpressing lines were all 100% (Figure 7C). However, after high-temperature treatment, the survival rates were 21.6% for WT, 7.7% for the *mterf5* mutant, and 74.3% and 56.1% for the two *SomTERF5* overexpressing lines, respectively (Figure 7D). These results showed that the survival rate of the *mterf5* mutant was significantly lower than that of the WT after high-temperature treatment, whereas the overexpressed *SomTERF5* lines exhibited significantly higher survival rates. Therefore, these findings suggest that overexpression of SomTERF5 enhances heat tolerance in *Arabidopsis*.

Additionally, it was observed that *SomTERF5* overexpression affects seed size in *Arabidopsis*, with the *mterf5* mutant displaying shorter pods compared to the WT. Conversely, the pods of the *SomTERF5* overexpressing lines were longer than those of the WT (Figure S2). Meanwhile, the seeds of the *mterf5* mutant were smaller than those of the WT, and the seeds from the overexpressing *SomTERF5* lines exhibited significant increases in size and weight (Figure S2). These findings show that the overexpression of the SomTERF5 gene can also improve pod and seed size in plants. These results indicate that mTERF5 and SomTERF5 may have functional similarity in *Arabidopsis* and spinach.

### 2.8. SomTERF5 Interacts with SopTAC2

To investigate the regulatory mechanism of *SomTERF5* under heat stress and during growth and development, the EXPLICT kinase prediction model was used to predict potential intercalating proteins of mTERF5, a homologue of SomTERF5. The results showed that mTERF5 could interact with pTAC2. The nuclear genome encodes the RNA polymerase (PEP)-specific cofactor, pTAC2 (PAP2), which mainly affects the formation of the plastid transcription complex [30,37].

**Figure 8:**
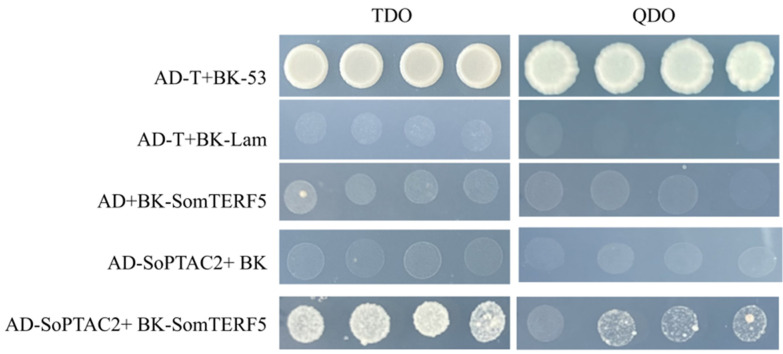
Validation of SomTERF5-SoPTAC2 interactions. pGADT7-SopTAC2 and pGBKT7-SomTERF5 plasmids co-transformed into *Saccharomyces cerevisiae* (Y2HGold) for interaction assessment. Negative (pGADT7-T/pGBKT7-LAM) and positive (pGADT7-T/pGBKT7-53) controls included to verify system validity and result reliability.

To ascertain whether SomTERF5 interacts with the SopTAC2 protein, yeast two-hybrid experiments were conducted. The coding sequences of *SomTERF5* and *SopTAC2* were fused to the pGBKT7 and pGADT7 empty plasmids, respectively, resulting in *pGBKT7-SomTERF5* and *pGADT7-SopTAC2* recombinant plasmids. Positive and negative controls were established using *pGBKT7-53/pGADT7-T* and *pGBKT7-Lam/pGADT7-T*, respectively. These plasmids were co-transformed into *Saccharomyces cerevisiae* (Y2HGold), plated on selective media, and incubated at 30 °C for two days. Monoclonal colonies were subsequently inoculated on three- and four-deficient media. The results demonstrated that yeast co-transformed with the recombinant plasmids proliferated on two-deficient media, while the positive control and the combination of *pGBKT7-SomTERF5* and *pGADT7-SopTAC2* also showed growth. In contrast, yeast co-transformed with the negative control and other recombinant plasmids exhibited no growth on three-deficient and four-deficient media (Figure 8). These findings suggest that the SomTERF5 protein lacks self-activating activity and that there is a direct interaction between SomTERF5 and SopTAC2 in yeast cells.

## 3. Discussion

### 3.1. Higher Homology Between Spinach SomTERFs and Arabidopsis mTERFs

The mTERF family was first discovered in animals, where it was localized in mitochondria and named mitochondrial transcription termination factor [12,23]. In animals, there are four mTERF members, while *Arabidopsis* contains a total of 35 *mTERF* genes. These genes have been classified into six major classes based on their localization and sequence differences [2,3]. Notably, the genomes of peanut, tomato, maize, and rice have been found to contain 17, 28, 31, and 48 mTERF members, respectively [18,19,20,21]. In this study, we identified 31 SomTERF members in the spinach genome through HMM and structural domain analysis (Table S1, Figure 1 and Figure 2). Interestingly, *Chlamydomonas reinhardtii* has only six members, and animals have four, indicating a significant expansion of the mTERF family in plants.

A notable observation is the distinct subcellular localization of mTERFs in plants, wherein they are found in both mitochondria and chloroplasts [23,24]. Functional studies have elucidated that the primary role of mTERFs is to regulate the expression of organelle genes. The expansion of this family in plants likely facilitates adaptation to environmental demands through precise regulation of gene expression in plant organelles [23,24,25,36]. A similar phenomenon has been observed in the PPR (pentatricopeptide repeat) protein family [38]. The protein homology between the 31 SomTERFs and the members of *Arabidopsis* mTERFs led to their designation as SomTERF1-SomTERF31 (Table S1) [2]. However, spinach and *Arabidopsis* differ by four members (SomTERF32-SomTERF35), which may warrant further investigation. The construction of a phylogenetic tree incorporating seven species (spinach, sugar beet, *Arabidopsis*, maize, *Chlamydomonas reinhardtii*, and humans), encompassing 168 amino acid sequences of mTERF proteins, resulted in the classification of all mTERFs into nine groups (Groups I-IX) (Figure 1). The distribution of gene numbers across these groups varied considerably, with Groups VI and IX containing more members, suggesting that these genes are more evolutionarily advanced. This phenomenon also appears in the genes of the same family in *Arabidopsis* [24], indicating that this evolutionary trend may be prevalent across different plant species.

### 3.2. SomTERF5 Affects Plant Heat Tolerance

In this study, 5-day-old seedlings of a WT, *mterf5* mutant, and overexpressed SomTERF5 in *Arabidopsis* plants were subjected to a high temperature, and their survival rates differed (Figure 7). Under normal conditions, the survival rates of the WT, *mterf5* mutant, and *SomTERF5* overexpressing lines were not affected. However, after high-temperature treatment, the survival rate of the *mterf5* mutant significantly decreased, but two *SomTERF5* overexpressing lines showed stronger resistance to heat. Thus, overexpression of the *SomTERF5* gene can enhance heat tolerance in *Arabidopsis*. This suggests that the mTERF family is involved in critical processes associated with plant growth and development, particularly regarding heat tolerance. It is highly possible that *SomTERF5* plays a substantial role in spinach’s response to heat stress. Therefore, further analysis of the downstream genes regulated by SomTERF5 is imperative to gain a deeper understanding of the molecular response mechanisms of SP75 spinach to heat stress. In *Arabidopsis*, mTERF5 can interact with pTAC6, which facilitates increased recruitment of pTAC6 to the PEP complex, thereby enhancing its transcriptional activation capacity [24,26]. In this study, we found that SomTERF5 and SoPTAC2 are reciprocal proteins through yeast two-hybrid experiments (Figure 8). The chloroplast nucleoid serves as a core region for the expression of photosynthetic genes and DNA metabolism, and its structural stability is a prerequisite for leaf survival under high-temperature stress. It has been shown that nucleoid-organizing proteins, such as WHIRLY1, maintain the topology of single-stranded DNA/RNA and coordinate the transcriptional activity of photosynthesis-related genes [39]. The interaction between SomTERF5 and SoPTAC2 discovered in this study (Figure 8) may regulate the assembly efficiency of RNA polymerase complexes, thereby maintaining the continuous expression of key genes like *rpoB* under high temperatures. This mechanism is similar to the regulatory function of rice mTERF14 in *rpoB* precursor processing [24,39], suggesting that plant mTERFs balance photosynthetic gene transcription and stress responses through evolutionarily conserved pathways. This finding not only provides new molecular targets for regulating heat tolerance in spinach but also reveals the potential multifunctionality of the SomTERF protein family in response to high-temperature stress.

## 4. Materials and Methods

### 4.1. Identification and Cloning of mTERFs Genes in Spinach

The spinach genome was downloaded from the SpinachBase website (http://www.spinachbase.org/; accessed on 5 June 2023) [3], and the HMM (Hidden Markov Model) file was downloaded, followed by the spinach protein library search of the acquired mTERF structural domains using HMMER software (v3.4), while the 35-member protein sequences of *Arabidopsis* thaliana were subjected to protein sequence comparison in the spinach protein library (BLASTp), with the E-value set to E-value < 1E^−100^ in BLASTp, and the obtained SomTERF candidates were analyzed using the NCBI-CDD (https://www.ncbi.nlm.nih.gov/Structure/cdd/wrpsb.cgi; accessed on 13 June 2023) and SMART (http://smart.embl-heidelberg.de/; accessed on 13 June 2023) for further mTERF structural domain validation, and 31 spinach mTERF members were finally identified. To verify the accuracy of the predicted mTERF sequences in the spinach genome database, we cloned the coding sequences (CDSs) of the mTERFs using SomTERFs primers (Table S2). The CDSs and protein sequences of the SomTERFs are shown in Table S3.

### 4.2. Phylogenetic Analysis of mTERFs in Different Species

A phylogenetic tree of mTERFs in spinach and six other plant species (i.e., sugar beet, *Arabidopsis*, common tobacco, woodland tobacco, maize, and rice) was developed. Of them, the protein sequences of 35 AtmTERFs, 31 ZmmTERFs, 33 OsmTERFs, 5 CremTERFs, and 4 HsamTERFs were obtained from previously reported articles. Other protein sequences were obtained from the *Arabidopsis* mTERFs Search Genome Data library (https://plants.ensembl.org/index.html; accessed on 13 June 2023). The phylogenetic tree of mTERFs was constructed in MEGA 7.0 using the neighbor-joining (NJ) method, with bootstrap replicates set to 1000, to analyze the evolutionary relationships between spinach and the homologous families of the other six species.

### 4.3. Analysis of Gene Structure, Functional Structural Domains, and Conserved Motifs

The exon–intron structure was analyzed using TBtools (v2.154) based on the genomic and CDS sequences of SomTERFs genes. Functional structural domains of SomTERFs were confirmed using NCBI-CDD. Protein sequence analysis was performed using MEME (https://meme-suite.org/meme/tools/meme; accessed on 13 July 2023) to identify the conserved motifs of SomTERFs and the maximum number of motifs was set to 10, and the results were visualized using TBtools [32].

### 4.4. Prediction of Promoter Cis-Acting Elements

The promoter sequence 2000 bp upstream of the SomTERFs gene start codon was extracted and analyzed for cis-acting regulatory elements using the PlantCARE database (http://bioinformatics.psb.ugent.be/webtools/plantcare/html/; accessed on 16 July 2023) [35], and the results are presented using TBtools. SomTERF promoters contain 10 categories of stress response elements, including heat response elements, cold response elements, drought response elements, light response elements, hormone response elements, damage response elements, anaerobic response elements, cell cycle regulation elements, circadian rhythm response elements, and cell metabolism response elements.

### 4.5. Plant Materials, RNA Extraction, cDNA Synthesis, and RT-qPCR

The heat-sensitive and heat-tolerant varieties of spinach, Sp73 and Sp75, were selected for gene expression analysis. Seeds were sown in trays containing a mixture of perlite substrate (1:1 ratio) and incubated in a plant growth chamber. Growth conditions were set at 22 °C light for 10 h/18 °C dark for 14 h and 60% relative humidity. The expression levels of *SomTERFs* genes were examined in different organs, including roots, stems, and leaves (i.e., first, second, and third leaves). Temperature stresses (low-temperature stress at 4 °C and high-temperature stress at 37 °C) were applied to seedlings at the six-leaf stage for 0, 2, 4, 12, and 24 h.

Total RNA was extracted from spinach leaves using TRIzol™ LS reagent (Ingenuity Life Technologies, USA). RNA was reverse transcribed to cDNA using the Prime Script reverse transcription kit, and the reverse transcription quantitative polymerase chain reaction (RT-qPCR) was performed using the SYBR Premix ExTaq II kit (Optimus Biologicals, China) with *SoARF* as the internal reference gene. Relative gene expression levels were normalized using the expression level of *SoARF*. Specific primer pairs were designed using the online tool Primer3 (http://bioinfo.ut.ee/primer3/; accessed on 12 November 2023) (Table S2). The RT-qPCR experiment was set up with three independent biological replicates. In addition, at least three technical replicates were performed for some representative genes in each set of RT-qPCR analyses. A two-sample *t*-test was used to evaluate statistical significance (Table S4). Each gene was normalized to the SoARF internal reference gene, and relative gene expression was calculated using the 2^−ΔΔCt^ method.

### 4.6. Subcellular Localisation of SomTERF5 and SomTERF17

SomTERF5 and SomTERF17 protein sequences without terminator CDS were constructed in pCAMBIA1300-GFP vector containing the CaMV35S promoter, respectively, and then the constructed vector and empty vector pCAMBIA1300-GFP were transformed into *Agrobacterium tumefaciens* strain GV3101, which was used to synthesize SomTERF5-GFP and SomTERF17-GFP fusion proteins through injection into tobacco leaves. The injected tobacco was incubated in the dark at 22 °C for 23 h and then under normal conditions (25 °C for 10 h light/20 °C for 14 h dark) for 33 h. The epidermal cells of the tobacco leaves from the above process were finally observed using a confocal laser scanning microscope (Olympus FV35, Tokyo, Japan).

### 4.7. Thermotolerance Analysis of Transgenic Arabidopsis

*Agrobacterium tumefaciens* strain EHA105 cells containing the pCAMBIA1300-35S-SomTERF5-GFP plasmid were transformed into Wild-type (WT) *Arabidopsis* seedlings using the inflorescence infiltration method to obtain seedlings overexpressing *SomTERF5*. The transformed seedlings were screened for three generations on 1/2 MS solid medium containing 40 μg/mL hygromycin. The expression level of the *SomTERF5* gene was assessed with RT-qPCR in several independent pure T3 generation lines using gene-specific primers (Table S2), and the lines with the highest expression were selected for heat tolerance analysis. WT, *mterf5* mutant, and *Arabidopsis* seedlings overexpressing *SomTERF5* were grown on 1/2 MS medium in a light incubator (16 h light at 22 °C/8 h dark at 20 °C, 75% relative humidity) for 8 days as a control treatment group. Heat-treated seedlings were first grown under control conditions for 5 days, then treated at 44 °C for 2.5 h, followed by 3 days under control conditions. At the end of the treatment, photographs were taken and recorded, and the survival rates (viable seedlings/all germinated seedlings) of the WT, *mterf5* mutant, and *Arabidopsis* seedlings overexpressing *SomTERF5* were calculated based on three independent biological replicates.

### 4.8. Yeast Two-Hybrid Experiments

The coding sequences of SomTERF5 and SopTAC2 proteins were fused with pGBKT7 and pGADT7 empty plasmids, respectively, to obtain pGBKT7-SomTERF5 and pGADT7-SopTAC2 recombinant plasmids. Meanwhile, pGBKT7-53/pGADT7-T and pGBKT7-Lam/pGADT7-T recombinant plasmids were used as positive and negative controls, respectively. Subsequently, co-transformation of *Saccharomyces cerevisiae* (Y2HGold) was conducted with the aforementioned plasmids, which had been coated with two-deficient medium and placed at 30 °C for two days of inverted incubation. Thereafter, monoclonal colonies were selected and inoculated onto three-deficient and four-deficient medium. The results were observed and photographed for the purpose of recording.

## Data Availability

All available data are contained within the article and Supplementary Materials.

## Supplementary Materials

The following supporting information can be downloaded at https://www.mdpi.com/article/10.3390/plants14111570/s1. Figure S1: Identification of the *mterf5* T-DNA insertion mutant in *Arabidopsis*; Figure S2: Overexpression of *SomTERF5* affects *Arabidopsis* pod and seed size; Table S1: Information on the mTERF gene family in spinach; Table S2: List of primers used in cloning and RT-qPCR analysis for SomTERFs; Table S3: List of the 168 mTERFs genes from spinach, sugar beet, *Arabidopsis*, rice, maize, *Chlamydomonas reinhardtii*, and humans; Table S4: Raw data used for Figure 4 and Figure 5 with significance analysis.
