# Supplementary material for: Genome-Wide Identification and Characterization of the mTERF Gene Family in Spinach and the Role of SomTERF5 in Response to Heat Stress
Source: Plants (Basel). 2025 May 22;14(11):1570. doi: 10.3390/plants14111570 (PMC12157027; doi:10.3390/plants14111570)
Supplement: Supplementary file 1 [file plants-14-01570-s001.zip › Supplementary Figures S1 and S2.pdf]

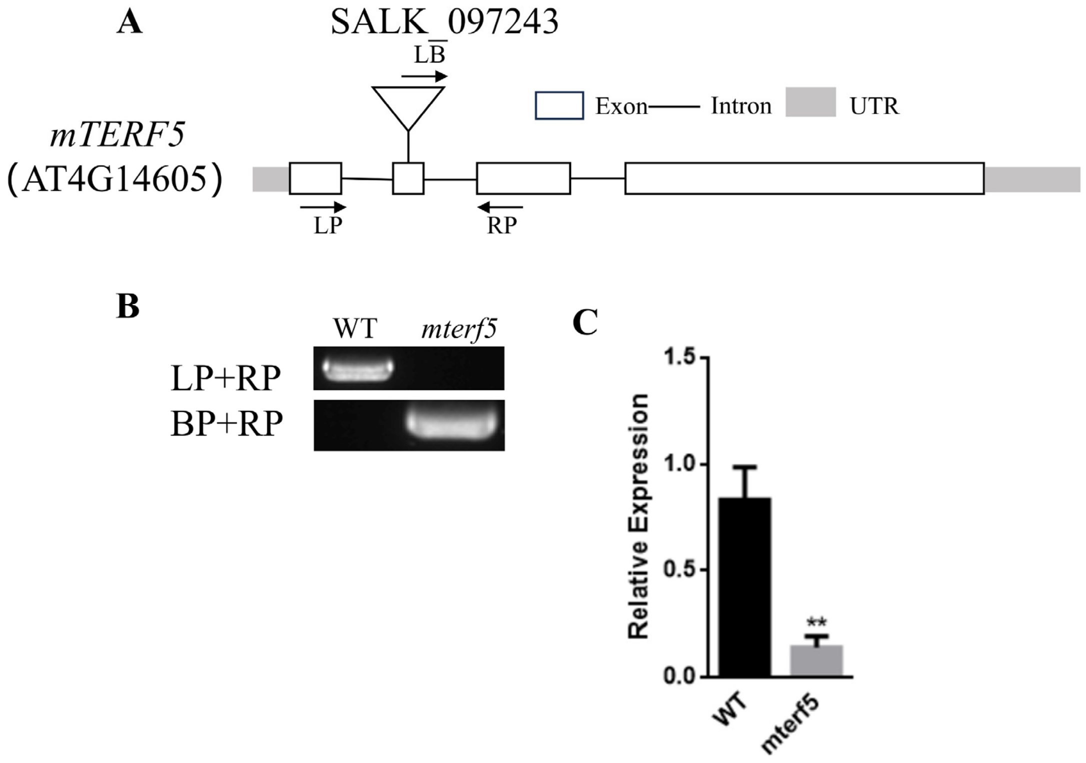

Figure S1. Identification of *Arabidopsis mterf5* T-DNA insertion mutant. (A) Schematic of T-DNA insertion site in *mterf5* mutant. (B) Gel electrophoresis image of *mterf5* mutant genotyping via three-primer PCR. (C) RT-qPCR analysis of *mTERF5* expression in *mterf5* mutant. Bars: mean ± SD. Data are shown as the means ± SD; $n = 3$; ** $p < 0.01$ based on Student's *t*-test.

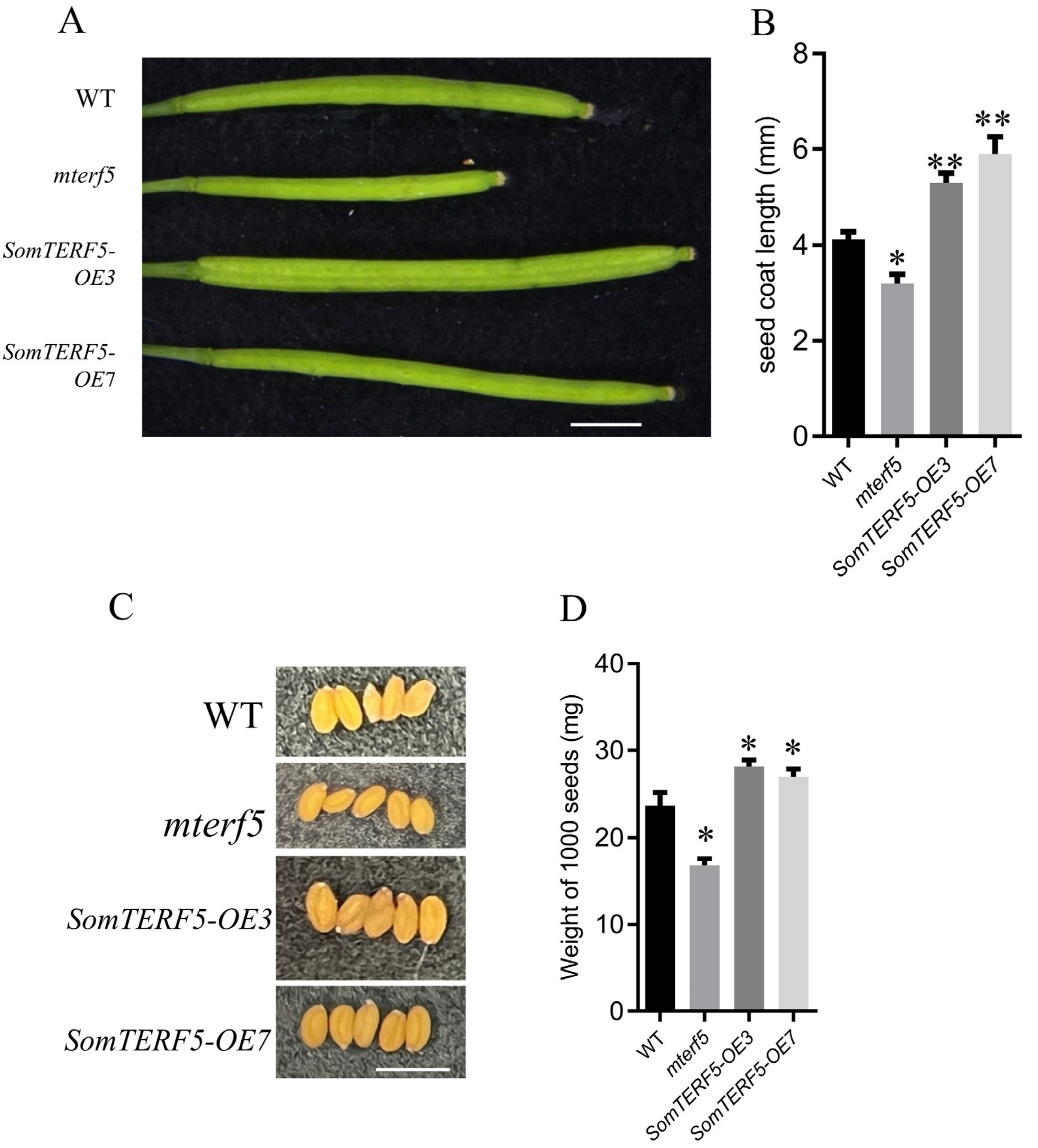

Figure S2. Overexpression of *SomTERF5* affects *Arabidopsis* pod and seed size. (A - B) Fruit pod size differences in WT, *mterf5* mutant, and *SomTERF5* -OE3/-OE7 overexpression lines. (C-D) Seed size of each *Arabidopsis* genotype with 1000-seed weight data, showing *SomTERF5* overexpression's effect on seed development. Bars: mean ± SD. Data are shown as the means ± SD; n = 3; * p <0.05 and ** p < 0.01 based on Student's t-test.
